# Make it grow: *Pseudozyma aphidis* extract promotes plant growth

**DOI:** 10.1093/plphys/kiag079

**Published:** 2026-04-29

**Authors:** Anton Fennec, Neta Rotem, Maggie Levy

**Affiliations:** The Institute of Environmental Sciences, Department of Plant Pathology and Microbiology, the Robert H. Smith Faculty of Agriculture, Food & Environment, The Hebrew University of Jerusalem, P.O.Box 12, Rehovot, Israel; The Institute of Environmental Sciences, Department of Plant Pathology and Microbiology, the Robert H. Smith Faculty of Agriculture, Food & Environment, The Hebrew University of Jerusalem, P.O.Box 12, Rehovot, Israel; The Institute of Environmental Sciences, Department of Plant Pathology and Microbiology, the Robert H. Smith Faculty of Agriculture, Food & Environment, The Hebrew University of Jerusalem, P.O.Box 12, Rehovot, Israel

## Abstract

Feeding the increasing global population requires higher agricultural yields while avoiding the use of harmful products. Beneficial micro-organisms provide an eco-friendly alternative to synthetic fertilizers and pesticides. We isolated a unique *Pseudozyma aphidis* strain demonstrating significant plant-protecting and growth-promoting activity on plants treated with live cultures. Here, we extracted and screened the most active plant growth-promoting fraction from *P. aphidis* and calibrated the dosage and application method. The fraction secreted by *P. aphidis* was applied to seeds and plants, thus eliminating the dependency on live colonies on the plants and leading to more consistent treatments with known extract concentration and repeatable results. We determined that the optimal application of the *P. aphidis* extract is seed coating combined with bi-weekly spraying at a concentration of 3 mg/mL. Tomato, corn, and melon plants treated with *P. aphidis* extract exhibited significantly enhanced germination rates shoot length, and biomass of treated tomato and melon plants. Plants treated with *P. aphidis* extract flowered 1 to 2 weeks earlier than the control and had increased yield. The treated tomato plants' fruit matured earlier, producing over 60% more ripe fruit than the control. The fruit also exhibited unexpectedly higher quality; it was firmer and had better flavor. We found that *P. aphidis* uses plant growth promotion mechanisms that include auxin-like molecules, siderophores and volatile molecule secretion. Our results highlight the potential of *P. aphidis* extract as an eco-friendly plant growth and crop-enhancing agent, reducing reliance on harmful agricultural inputs.

## Teaser

The bioactive fraction secreted by *Pseudozyma aphidis* enhances growth, yield, and crop quality.

## Introduction

The rapid growth of the global population and the constant demand to increase the agricultural yield require an intense use of fertilizers and pesticides. Fertilizers are a major source of nitrous oxide (N_2_O), which is considered a greenhouse gas contributing to global climate change (Rochette et al. 2008). A study of ice cores by Australia's National Science Agency revealed a rapid atmospheric buildup of N_2_O in the past 2000 years (CSIRO, 2022). Extensive fertilization also increases the rate of emerging dead zones and leads to soil and water pollution (Diaz and Rosenberg 2008; Khan et al. 2018). To paraphrase Oscar Wilde, either the environment goes or we do (Schwartz 2008). Thus, there is a necessity to shift from intense use of synthetic fertilizers (SF) and pesticides toward more sustainable alternatives.

Beneficial micro-organisms (BMOs), including plant growth-promoting fungi (PGPF), can provide an environmentally friendly solution that expedites reduced application of SF and plant growth-promoting (PGP) products (Yadav et al. 2020). The most common BMOs PGP modes of action include the following: phosphate solubilization, nutrient uptake, plant protection, stress tolerance, phytohormones, and nitrogen fixation (Singh 2018). Some BMOs can produce the phytohormone indole acetic acid and other auxin-like molecules (Chung et al. 2003; Sun et al. 2014). Additionally, glycolipid surfactants produced by some BMOs have shown a promising PGP activity (Marchut-Mikołajczyk et al. 2021). The PGP effect of BMOs is often demonstrated in the form of increased plant biomass and enhanced chlorophyll production (Nimsi et al. 2023).

Although some BMOs were found capable of enhancing plant growth, thereof treatments may reduce the crop quality parameters such as total soluble solids (TSS), an indicator for sugar and titratable acid (TA), a sourness indicator (Mostafa and Abdel-Salam 2018). Additional parameters that are often influenced by PGP treatments enhancing the crop yield also include titratable nitrogen (N), which acts as an indicator of nutritional value and produces firmness (Iyer et al. 2012). Produce flavor is another major quality parameter that may suffer a drawback from the aforementioned treatments (Tandon et al. 2003; Noceto et al. 2021). In fact, it has become one of the major complaints among consumers that the taste of agricultural produce has become bland over time (Estabrook 2012; Quick et al. 2022).

Although the potential effectiveness of BMOs on commercial crops was demonstrated, there are complications in their application in a large-scale industry and agriculture (Vejan et al. 2016; Katsenios et al. 2021; Muñoz-Carvajal et al. 2023). Various BMO species require diverse conditions. Hence, the application of live BMOs is somewhat limited by geography and climate (Nicot, 2011). Thus, there is an uncertainty of how the BMOs are established on the host plants. Furthermore, there are gaps in knowledge and regulation of how persistent their beneficial activity might be under different conditions (Sundh et al. 2021; Kou et al. 2024). Additionally, the upscale process from research to industrial application in sustainable agriculture still suffers from gaps in knowledge and wherewithal (Muñoz-Carvajal et al. 2023). The most common approach for establishing a long-lasting BMO growth on plants is by repeated application of new BMO inoculum (Vejan et al. 2016; Sundh et al. 2021; Kou et al. 2024). A study by Metwally et al (2022) on fungal extracts from Trichoderma cultures has shown that C-phycocyanin from the extract enhanced tomato germination and seedling growth (Metwally et al. 2022). Our approach takes us in a similar direction. We extract and apply the eco-friendly PGPF-based compounds instead of live cultures. Thus, we eliminate the need to maintain the BMO in the field and create a more repeatable and uniform functionality.

Agro-formulants, both biological and synthetic, such as fertilizers, pesticides, herbicides, and plant growth enhancing products are applied by several common methods (Knowles 2012). The most common practices for their application include irrigation, spraying, seed coating, or direct application to the soil (Girardin et al. 2000). Selecting the optimal application method requires matching the suitable medium for the substance and optimizing it for best performance and cost efficiency (Girardin et al. 2000; Knowles 2012). Alas, to date, many formulants are gravely used in sub-optimized manner that leads to higher costs and environmental impact (Girardin et al. 2000; Knowles 2012). We examined the most suitable application methods of our *Pseudozyma* extract that will be suitable for the substrate and of efficient activity.

Most of the studies on PGPF are conducted on arbuscular mycorrhizal fungi (AMF), while epiphytic and endophytic fungi are studied to a lesser extent (Bacon and White 2016; Elsherbiny et al. 2023). The preponderance of these studies is limited to juvenile plants. Hence, the information on the PGPF effects on mature plant physiology, yield, and crop quality is scarce (Nimsi et al. 2023). According to our survey, up to March 2025, there are only 1620 entries of *Pseudozyma aphidis*. Analysis of these entries has shown that only 5% of these studies are focused on the PGP aspects of *Pseudozyma*. Most of them are strictly focused on bioactive surfactants.

*P. aphidis* is a yeast-like dimorphic fungus related to the phytopathogenic fungus *Ustilago*. Unlike its relative, *P. aphidis* is not only non-pathogenic but also beneficial to plants (Barda et al. 2015; Gafni et al. 2015). We isolated a unique *P. aphidis* strain that has shown significant antifungal and antibacterial activity in our previous studies (Srivastava et al. 2021). *P. aphidis* exhibits biocontrol activity against powdery mildew and *Botrytis cinerea* (Buxdorf et al. 2013; Gafni et al. 2015; Calderón et al. 2019). Tomato and cucumber plants treated with live *P. aphidis* cell culture expressed enhanced plant growth (Shoam et al. 2022). Our early work on *P. aphidis* extract has demonstrated plant protective qualities of *P. aphidis* secretions, but the PGP aspect was not characterized in depth.

In some cases, PGP treatment has shown promising results in one crop but no effect or even negative effects on other crops. This was demonstrated by Andresen and Cedergreen (2010) who found an extract that promoted strawberry growth and yield but had a negative effect on barley. Here, we selected crop plants that represent agriculturally important crop plant families. Cereals are the largest staple food crops worldwide that incorporates maize (corn), rice, wheat, etc. The annual global production value of cereals was estimated at over 800 billion USD between 2012 and 2013 and it keeps increasing in both demand and value (IFAD, 2017; Schreinemachers et al. 2018). Corn is one of the major cereals that is used for fresh and processed human and animal food in addition to its use as fuel material (Erenstein et al. 2022). We selected corn for our study as a representative of the cereal plant family. Cucurbits are an important crop plant group that is also considered a staple food for its nutritional and economic value. The cucurbit family includes important crops such as pumpkins, cucumbers, melons, and watermelons, and their global market in 2012 was estimated at over 65 billion USD (IFAD, 2017). We selected melon for our experiment to represent the cucurbit family. The Solanaceae plant family is of major importance and includes tomatoes, potatoes, bell peppers, etc. In 2012, the global tomato market alone was estimated at over 90 billion USD/year (IFAD, 2017). Tomatoes are also well sequenced and often utilized as a model organism for both PGP and plant protection studies (Arie et al. 2007; Kimura and Sinha 2008).

Several *Pseudozyma* species have shown PGP activity, mostly in seedlings and juvenile plants. *Pseudozyma antarctica* produces and secretes itaconic acid (methylidene succinic acid) that increases plant nutrient uptake (Levinson et al. 2006; Zhao et al. 2022). Some *P. aphidis* isolates were found to produce ammonia (NH_3_) and auxin. Several PGP-capable isolates possess calcium phosphate tribasic solubilizing activity. Thus, they can enhance nutrient uptake of the host plant (Fu et al. 2016). For the most part, studies on the *Pseudozyma* PGP and biocontrol effects of live cultures and extracts were conducted on several non-industrial model cultivars. Yet, their focus remained on the biocontrol aspects (Golubev et al. 2001; Metwally et al. 2022). As the bulk of the work focused on plant protection, we extended our study to the PGP activity aspect of *P. aphidis.*

To identify the extract with PGP function without interference of direct biocontrol activity, the extracts need to be screened for the absence of pathogen inhibition. Our previous studies have shown the antibiotic activity of *Pseudozyma* extract against the pathogenic fungus *B. cinerea* and the bacterial pathogen *Pseudomonas syringae* (Srivastava et al. 2021). Often, the plant pathogen *Agrobacterium tumefaciens* is also used as a model organism for antibacterial activity screening (Robinette and Matthysse 1990; Jailani et al. 2022). We decided to validate our PGP extract's lack of antibiotic activity against the phytopathogens *B. cinerea* and *A. tumefaciens* due to their relevance as common plant pathogens.

Our *P. aphidis* extract acts as an eco-friendly PGP agent that increases plant growth, biomass, and also induces early flowering. Furthermore, the plants treated with our PA extract produced more yield and were of higher quality. The data of this study is focused on the characterization of the *P. aphidis* extract activity on growing crop-bearing plants relevant in agriculture, from germination to harvest, including the effect on crop quality. These results demonstrate the potential of compounds secreted and extracted from *P. aphidis* to contribute to green agriculture and global food security.

## Results

### PA extract pharmacology

**Figure 1:**
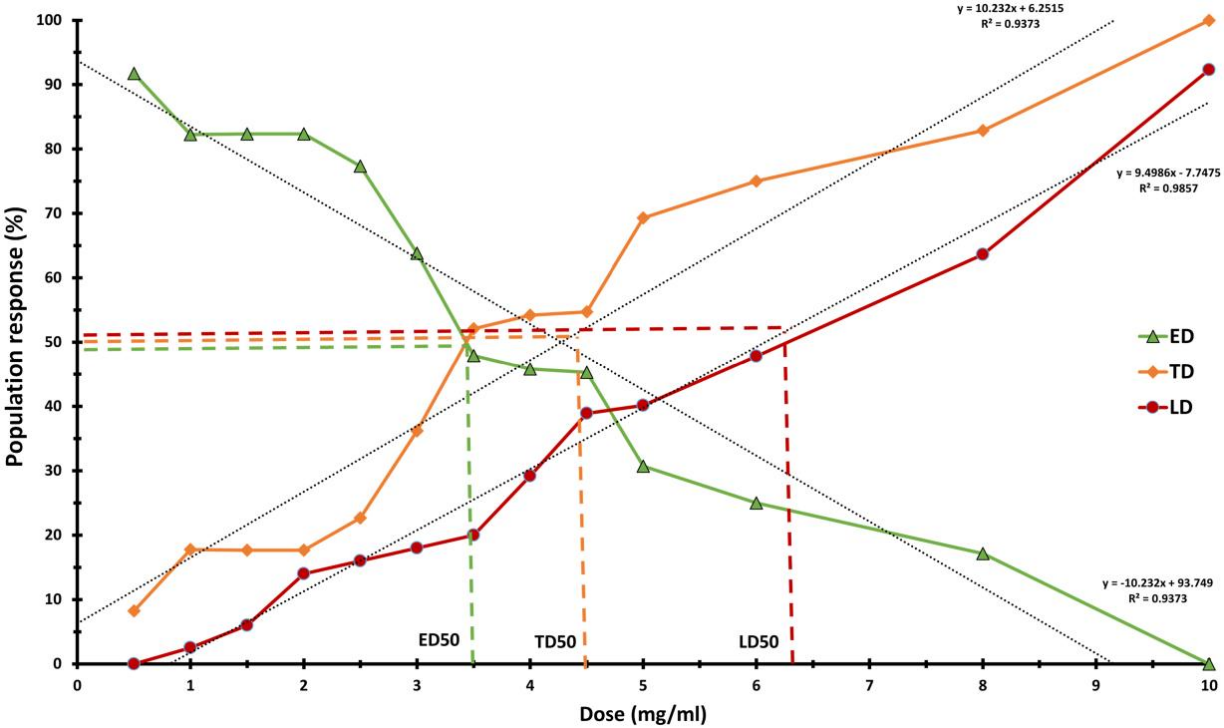
Pharmacology and optimal extract dose. The effective dose (ED, triangles), the toxic dose (TD, diamonds), and the lethal dose (LD, circels). LD was determined by the survivability rate of germinating seeds and seedlings over a duration of up to 10 days post-germination. The ED is derived from the portion of the seed population that has successfully germinated, based on survivability metrics and the beneficial influence on both root and shoot elongation, standardized using the log method. The TD is established as an inverse trend of ED (see Methods). LD50 is the dose that proves fatal to 50% of the population, whereas TD50 refers to the dose that exerts toxic effects on 50% of the population, and ED50 is the dosage that induces a positive response in 50% of the population. Dotted lines represent the population response trends, the fine dotted lines represent the correlation between dose and population response. The dashed lines represent the following values: LD50 = 6.3 mg/mL, TD50 = 4.5 mg/mL, and ED50 = 3.5 mg/mL.

This study aimed to extract and isolate the PA-secreted bioactive PGP fraction to elucidate its biological activity through application to crop plants. We evaluated the crude extract fractions derived from cultures cultivated in Murashige and Skoog (MS) and Potato Dextrose Broth media across a large range of hydrophobic to hydrophilic affinity and determined that the optimal extract conditions are MS medium cultures extracted with hexane:acetone in a ratio of 70%/30% (v/v) (Table S1). We assessed our fraction for its antimicrobial activity and found that it did not exhibit any direct inhibitory effects against *B. cinerea* and *A. tumefaciens* (Table S2). Thus, we successfully separated the compounds associated with PGP activity from those with antibiosis activity. Pharmacological tests of the PGP extract on Col-0 seeds and seedlings indicated that the LD50 of our extract is 6.3 mg/mL, the TD50 is 4.5 mg/mL, and the ED50 is 3.5 mg/mL. We decided to apply the extract at a concentration of 3 mg/mL, which facilitates approximately 70% of ED with consistent optimal performance (Fig. 1).

### Application method screening

The evaluation of an optimal application method utilizing a singular parameter with multiple effects may be highly inaccurate. A multivariate correlation matrix between PGP parameters derived from all the extract application methods (seed coating, spray, and combined) and a control was used for a selection of parameters and their combination that significantly contributed to their efficacy. Parameters exhibiting high correlation (*r*^2^ value) were selected as determinants for PGP activity of the optimal application method (Fig. S1a and b). Thus, we concluded that both seed coating combined with bi-weekly spraying and seed coating alone have exhibited the best performance. The combined approach of seed coating and bi-weekly spray had a slight edge in regards to crop ripening (Fig. S1c).

### PA extract effect on juvenile plant germination and growth parameters

**Figure 2:**
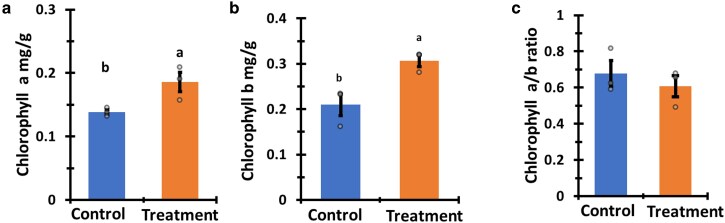
Juvenile plants' chlorophyll concentration. Chlorophyll a and b content and ratio of a/b chlorophyll of 5-week-old tomato plants treated with *P. aphidis* extract (treatment—right) and untreated (control—left). **(a)** Chlorophyll a, **(b)** Chlorophyll b, and **(c)** Chlorophyll a/b ratio. The bars are mean and the error bars represent standard error, connecting letters over the bars represent statistical significance (Student's *t*-test, *α* = 0.05).

To characterize the influence of *P. aphidis* extract on crop plants (tomato, corn, and melon) during their juvenile phases, we assessed the germination rates, leaf quantity, leaf length, shoot length, and chlorophyll concentration of treated 5-week-old plants in comparison to control plants (Table 1, Fig. 2).

**Table 1:** Physiological parameters of corn, melon and tomato plants following *P. aphidis* extract treatment.

Plant	Group	Germination (%)	Shoot length (cm)	Leaf length (cm)	Number of leaves
Maize	Control	92.16 ± 1.09 b	15.86 ± 0.43 b	47.8 ± 2.48 b	6 ± 0
	Treatment	100 ± 0 a	20.2 ± 2.15 a	55.46 ± 0.89 a	6 ± 0
Melon	Control	86.7 ± 2.23 b	22.46 ± 1.26 b	8.54 ± 0.34 b	3.1 ± 0.16 b
	Treatment	93 ± 1.09 a	25.6 ± 1.48 a	9.29 ± 0.37 a	4.1 ± 0.16 a
Tomato	Control	77.7 ± 7.8 b	28.3 ± 0.7 b	11.7 ± 2.4 b	9 ± 0.7
	Treatment	96 ± 2.6 a	31.3 ± 0.5 a	17.9 ± 2.9 a	9.2 ± 0.9

The mean and standard error are accompanied by connecting letters that represent significant differences calculated with Student's *t*-test, α=0.05; *n* = 100 (germination), 20 (shoot length, leaves length, and number of leaves).

All the plants treated with *P. aphidis* extract have shown a significantly higher germination rate compared with the control (Table 1). Treated tomato seeds demonstrated the highest increase in germination (18%), while treated corn and melon seeds germination increased by ±7% (Table 1). The coated seeds of all the treated plants germinated 2 to 3 days sooner than the corresponding control seeds (Table S3). Treated juvenile plants that reached a developmental stage of 5 weeks exhibited significantly elongated shoots and leaves (Table 1). Tomato shoots were 9.5% longer, melon shoots were 12.2% longer and corn shoots were 21.4% longer than those of the control (Table 1). Treated melon plants had 24.3% more leaves compared to the control. No effect on the number of leaves was observed on corn and tomato plants (Table 1). Treated melon leaves were longer by 8%, corn leaves by 13.8% and tomato leaves were 34.6% longer in comparison to the control. The chlorophyll a and b content was significantly higher by 46 and 97 μg/g, respectively, compared to the untreated control plants (Fig. 2a and b). There was no significant change in the chlorophyll a to b ratio following treatment (Fig. 2c).

### Treatment effect on flowering timing

**Figure 3:**
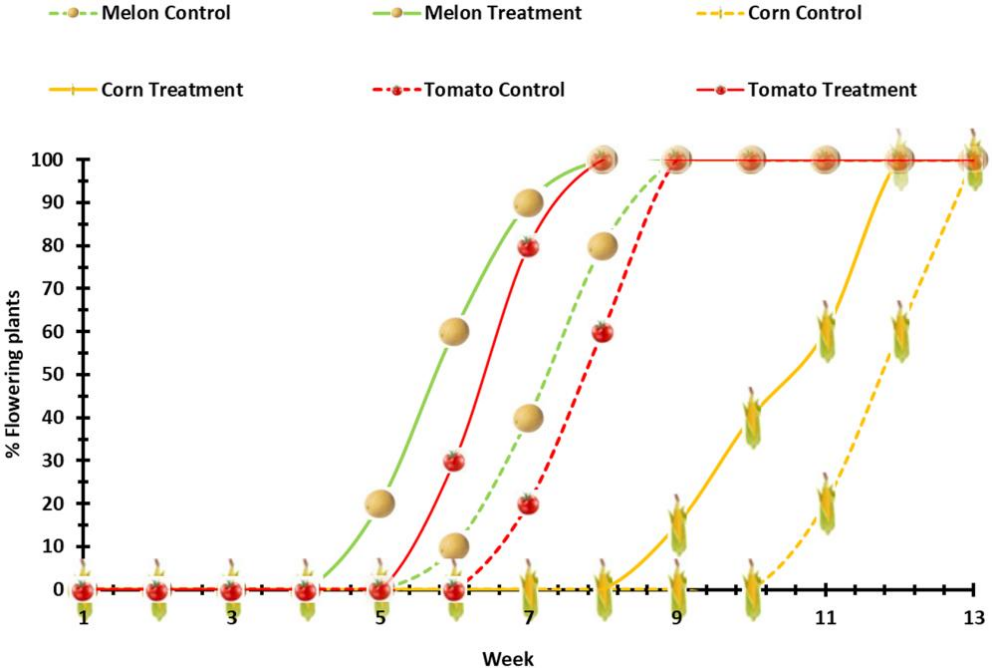
Flowering time from start to full bloom. Time stamps of plants of all cultivars (melon, tomato, and corn), continuous lines represent treated plants, and dashed lines represent the control plants. Data are shown in weeks from flower formation on the plants to full blossom of all plants for each cultivar. The results are shown in % of the flowering plants of each cultivar vs all the plants per given cultivar.

Evaluation of flower development and time to full bloom is shown in Fig. 3. The treated plants reached full bloom 1 to 2 weeks sooner than their control counterparts (Fig. 3). The treated melon and tomato plants initiated flowering and reached full bloom 1 week earlier than the control plants. The treated melon plants started flowering 2 weeks prior to the control plants, but reached full bloom only one week sooner (Fig. 3).

### Treatment effect on mature plant growth and yield

**Figure 4:**
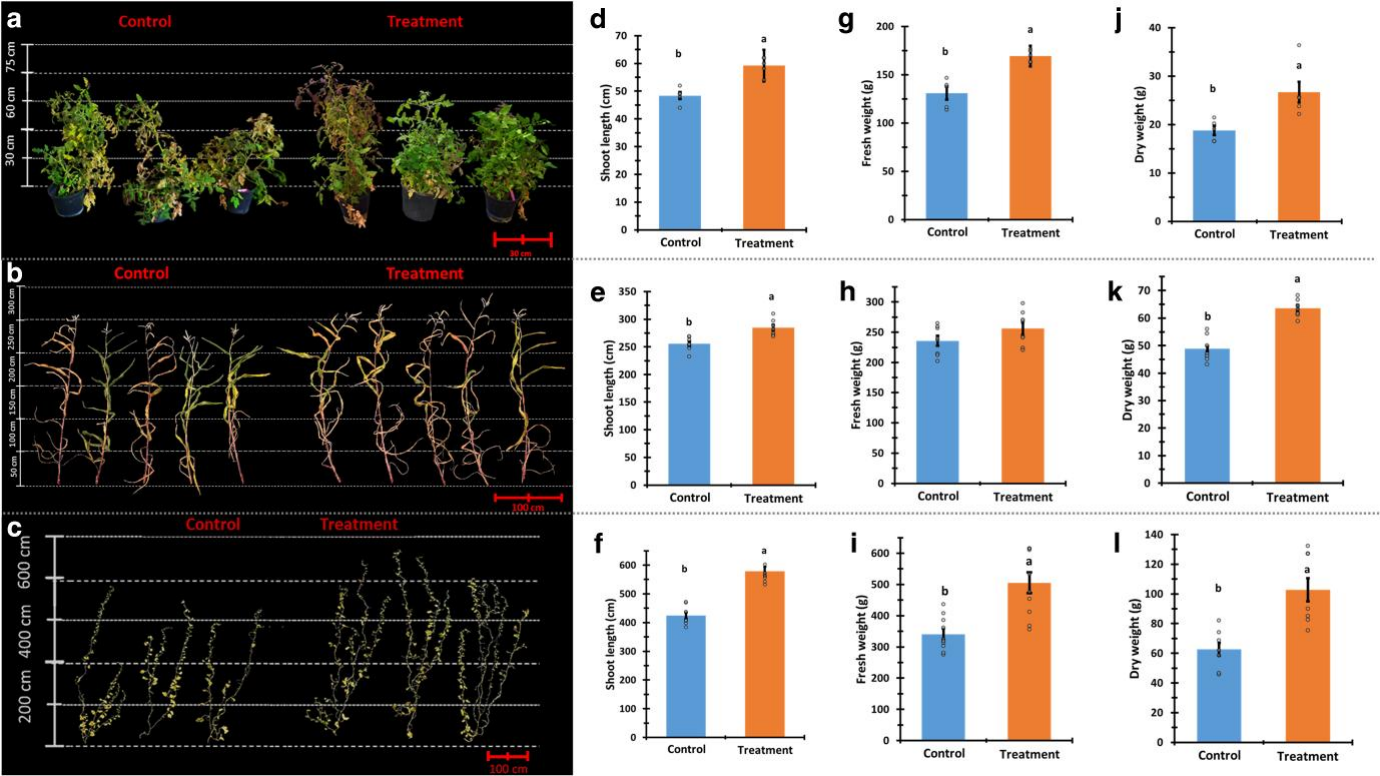
Plant height and biomass. Representative tomato, corn, and melon plants' images with scale measure (a–c accordingly). Shoot length of the plants of all the cultivars: d—tomato, e—corn, and f—melon. The fresh and dry weight of the plant stalks: tomato—g & j, corn—h & k, melon—i & l (accordingly). Left bars represent the mean of the control group and the right bars represent the treated plant group. Mean and standard error are accompanied by connecting letters over the bars that represent a significant difference calculated with Student's *t*-test, *α* = 0.05; for shoot length *n* = 5 (tomato plants) and 7 (corn and melon plants). For fresh and dry weight *n* = 5 (tomato), 8 (corn), and 10 (melon plants). The images background was digitally extracted for comparison.

We examined the effect of *P. aphidis* extract treatment on plant growth upon plant maturation. Assessment of plant growth was based on the following parameters: plant size, fresh and dry weight, and yield of the 3 crop plants: tomato, melon, and corn. Shoots of mature plants treated with *P. aphidis* extracts were significantly longer across all the crop plants compared to the untreated control. The shoot height of treated tomato plants was higher than the control by 22%, melon by 36%, and corn by 11.4% (Fig. 4a–f). The fresh and dry biomass of the treated plants was significantly higher than that of the control (Fig. 4g–i). The fresh weight of treated tomato plants was higher by 29.4% and the melon was 48.5% higher than the control (Fig. 4g and i). No significant effect on the fresh weight of corn plants was observed (Fig. 4h). The dry weight of treated plants of all the crops has substantially increased; tomato by 42.7%, corn by 30.1%, and melon by 64.2% in comparison to the control (Fig. 4j–l).

**Figure 5:**
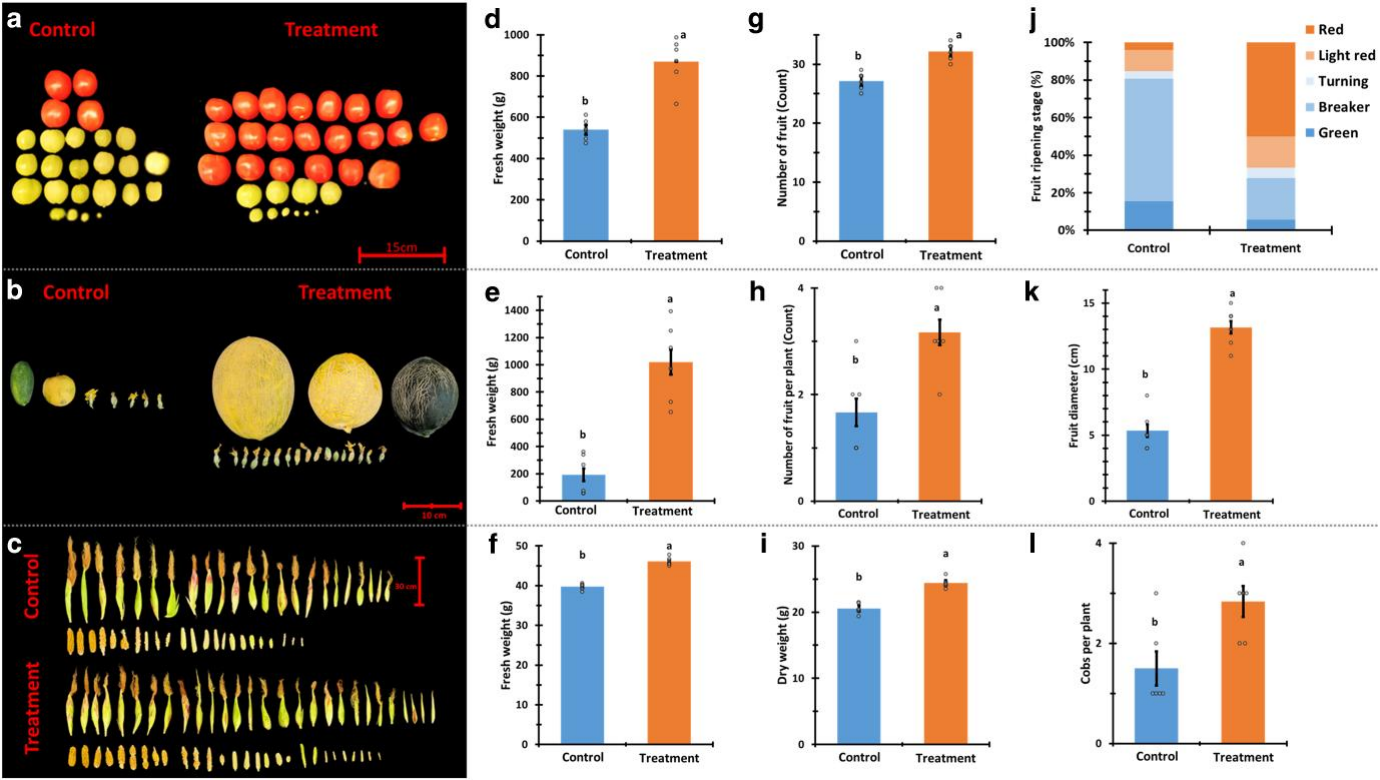
Crop, yield, and ripening. Representative image of tomato fruit (per plant), melon (per plant), and corn cobs (collected from 10 plants) with a scale (a–c accordingly). The fresh average weight of tomatoes per plant—d, melon fruit—e, and 100 corn kernels—f. Number of fruit from tomato—g, melon plants—h, and 100 corn kernel dry weight—i. Tomato ripening fruit by stage represented in percentage from the total number of fruit (ranking system in the methods section)—j, average melon fruit size expressed in diameter—k, and number of cobs per corn plant—l. Blue bars represent the mean of the control group and the orange bars represent the treated plant group. The mean and the standard error are accompanied by connecting letters that represent a significant difference calculated with Student's *t*-test, *α* = 0.05; for all the parameters, *n* = 6. The images background was digitally extracted for comparison.

Once the plants reached maturity, we characterized the effect of *P. aphidis* extract treatment on the yield. The harvested crops were examined for total yield and ripening parameters (Fig. 5). Treated plants produced distinguishably higher yield in comparison to the control. Treated tomato plants produced fruits that weighed 61.4% more than the control, and their yield increased by 18.4% more tomatoes per plant. The fruit of the treated tomato plants ripened faster (3 times more ripe fruit), and were more uniform than those of the control (Fig. 5a, d, g and j). The treated melon plants produced fruit weighing 429.4% more than that of the control and larger in diameter by 148%. An average treated melon plant produced 90.3% more fruit in various development stages and more ripe fruit per plant than the control (Fig. 5b, e, h and k). The average fresh and dry weight of 100 kernel corns of treated plants was 15.9% and 19% higher than the control, respectively. The treated corn plants also produced 88.6% more cobs per plant (Fig. 5c, f, i and l).

**Figure 6:**
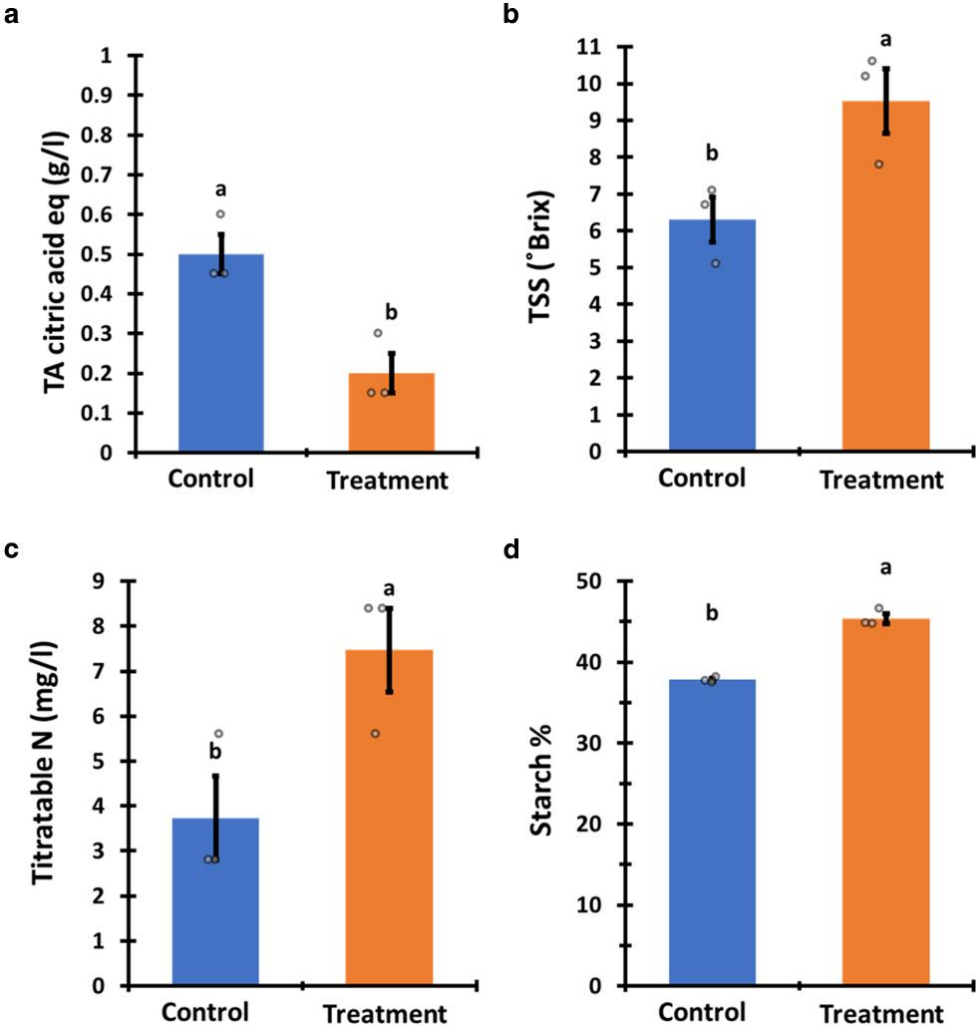
Melon and corn quality. Crop quality was expressed in melon TSS—a, TA—b, titratable nitrogen content (N)—c and corn starch (%)—d. The left bars represent an average of the control group and the treated plant group is shown on right bars. Mean and standard error accompanied by connecting letters over the bars represent a significant difference calculated with Student’s test, *α* = 0.05; *n* = 3.

**Figure 7:**
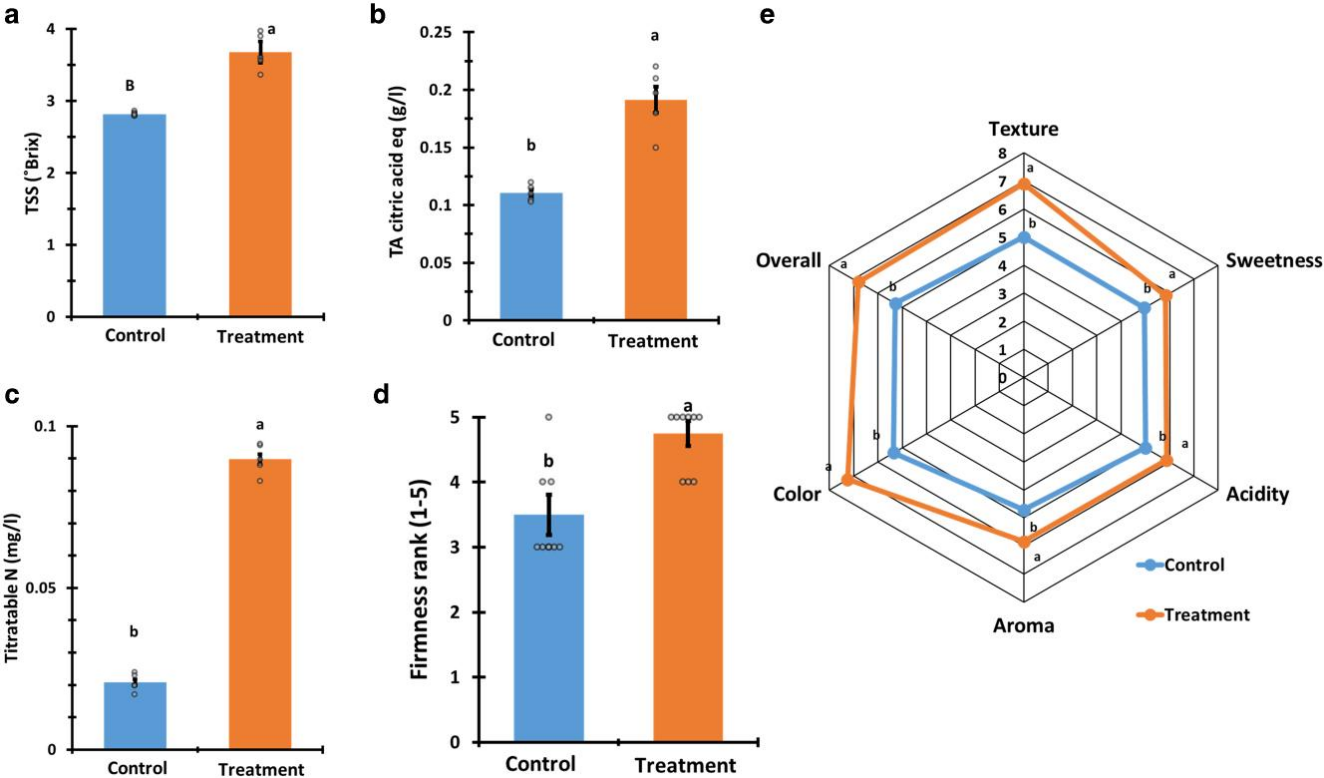
Tomato fruit quality. Fruit quality was expressed in TSS—a, TA—b, titratable nitrogen content (N)—c, fruit firmness index (as described in the Methods section)—d, and a sensory test (described in Methods)—e. The blue bars in panels a–d and lines in panel e represent the mean of the control group and the orange bars in panels **a–d** and lines in panel **e** represent the treated plant group. Mean and standard error accompanied by connecting letters over the bars in panels a–d and adjacent to the lines in panel e that represent significant difference calculated with Student’s *t*-test, *α* = 0.05; for TSS, TA, and N, *n* = 5. For firmness and taste test, *n* = 9 and 32, accordingly.

Subsequently, we examined the effect that *P. aphidis* extract treatment has on fruit crop quality (Figs. 6 and 7). Fruit of the treated melon plants had a significantly higher TSS (50%), soluble nitrogen content (201%), and TA that is 2.5 times lower than that of the control (Fig. 6a–c). Corn starch content from kernels from the treated plants was higher by 20% compared to the control (Fig. 6d).

Fruit of the treated tomato plants had a significantly higher TSS (30.6%), TA (72.7%), and soluble nitrogen content (345%) than the control (Fig. 7a–c). Fruit firmness from the treated plants was also significantly higher by 35.7% (Fig. 7d). A sensory taste test was performed to assess the impact of the treatment on tomato fruit flavor (Fig. 7e). Fruit of the treated plants received significantly higher testers score across all the assessed parameters (sweetness, acidity, aroma, color, and texture). The color and texture scores were higher by 2 ranks. The sweetness, acidity, and aroma scores were higher by 1 rank. The overall preference score of tomatoes of the treated plants was 2 ranks (20%) higher in comparison to the control fruit (Fig. 7e).

### *P. aphidis* extract possible PGP mechanisms

**Figure 8:**
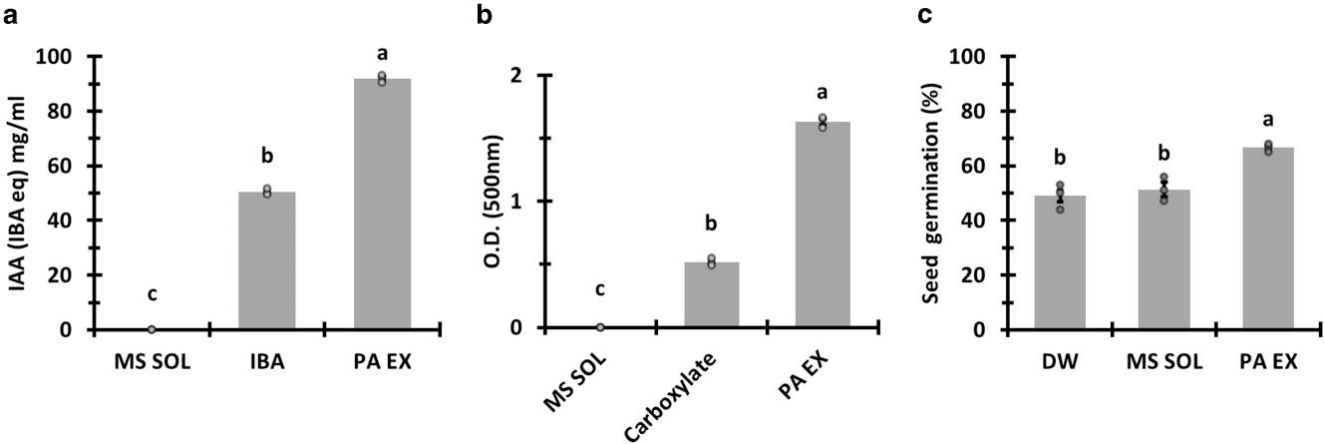
IAA, siderophores and volatiles affect seed germination. IAA **(a)**, siderophores concentration **(b)**, and the *P. aphidis* extract (PA-EX) volatiles effect on seed germination **(c)**. MS culture media extracted with solvent (MS SOL). Indole butyric acid (IBA) was used as positive control for IAA analysis **(a)** and carboxylate as a positive control for siderophore analysis **(b)**. The bars represent mean ± standard error and the connecting letters over the bars represent statistical significance (Student’s *t*-test, *α* = 0.05, *n* = 3 averages of 3 experiments for IAA and siderophores and for volatiles 3 trays containing 20 seeds per treatment).

We examined several possible *P. aphidis* extract treatment modes of action regarding its PGP activity. The extracted fraction contained 92 mg/mL auxins and auxin-like compounds (Fig. 8a). In addition to auxins, the extracted fraction was also found to contain siderophores (Fig. 8b). The volatile activity of our *P. aphidis* extract was tested on seed germination. We found a 15% increase in germination of seeds incubated with the *P. aphidis* extract compared with the controls (Fig. 8c).

## Discussion

Although *P. aphidis* is known to have biocontrol and PGP activity, there is scarce information on the PGP aspect, the application, effects on yield, and crop quality are not well characterized. In previous investigations conducted within our laboratory, treatments utilizing live *P. aphidis* cultures enhanced plant growth and increased the yield of M-82 tomato plants (Shoam et al. 2022). Several observations concerning the PGP activity of *Pseudozyma* species by various research groups are reported in the literature. However, often they are not focused on *Pseudozyma* and it is clustered with other PGP capable micro-organisms (Levinson et al. 2006; Boekhout 2011; Fu et al. 2016).

In this research, we aimed to extract and isolate the bioactive PGP fraction secreted by *P. aphidis*, to characterize its activity by application on crop plants and examine some of the mechanisms involved in the PGP activity of our *P. aphidis* extract. Our findings demonstrate that *P. aphidis* is producing and secreting the phytohormone auxin and auxin-like molecules in higher concentrations than those found in native M82 tomato tissue (Fig. 8). Our results correspond to the findings of Muthukrishanan et al. (2024) and Ramos-Garza et al. (2023), who found that some *Pseudozyma* species possess the capacity of auxin biosynthesis (Ramos-Garza et al. 2023; Muthukrishanan et al. 2024). Another PGP mechanism utilized by some BMOs, including PGPF and demonstrated mostly in AMF, is the production of siderophores (Mishra et al. 2016; Zhu et al. 2025). We found that *P. aphidis* is producing and secreting siderophores in order to chelate iron from the environment as part of its PGP ability (Fig. 8b).

The optimal conditions for extract preparation to investigate PGP activity were an extract from MS media culture, extracted with hexane:acetone (Table S1). Our screening methodology, coupled with the PGP activity bioassay, has demonstrated that the extract we selected did not exhibit any antibiosis properties *in vitro* (Table S2). Thus, we successfully separated the compounds responsible for PGP and those that have antibiosis activity. This approach allowed us to reduce the interference of antibiotic activity and to characterize the PGP activity of extracted compounds secreted by *P. aphidis*. Bioactive molecules are known to have a positive effect in low concentrations and are lethal in high concentrations (Puga-Freitas et al. 2012). We determined that the optimal beneficial concentration of *P. aphidis* extract is in a range of 3 to 4.5 mg/mL (Fig. 1). The optimal application method as we determined, is seed coating combined with bi-weekly spraying (Figure S1). These application techniques are consistent with the standard agricultural application practices (Verkleij 1992). Seed coating and spraying treatments were done with 3 mg/mL extract concentration and followed by measuring the physiological effects on crop plants.

We observed a significant effect on plant growth and yield of all the examined crops following treatments with the *P. aphidis* PGP fraction. The treated plants exhibited an earlier flowering time by 1 to 2 weeks than the control in both flowering initiation and in the time of full bloom. While corn flowered 2 weeks ahead of its control, tomato and melon plants flowered 1 week ahead of the control (Fig. 3). All the treated plants reached full bloom 1 week sooner than the control. We documented and report in this study an early flowering induced by *P. aphidis* or its extract.

Prior research on PGP focused mainly on AMF, with data supporting early flowering induction by mycorrhiza fungi (Sohn et al. 2003). Early flowering can facilitate early fruit set and development that can potentially shorten the growing season and might have a significant impact on fast-growing crops (Van Nocker and Gardiner 2014). Our findings indicate that *P. aphidis* extract serves as an effective agent for promoting early flowering, fruit set and development, particularly in melon plants. All crop plants treated with *P. aphidis* extract exhibited significantly increased shoot lengths compared with the controls. Treated plants displayed enhanced shoot growth ranging from 10% to 36% (Table 1, Fig. 4d–f). Mature melon plants exhibited the best response to the treatment with a 36% increase in shoot length compared with the controls. Mature corn and tomato plants also experienced significant increases in shoot length due to *P. aphidis* extract treatment, albeit to a lesser degree of 11% and 22% (Fig. 4b, e, h, k and a, d, g and j).

AMF with PGP activity are recognized for their role in enhancing chlorophyll content and photosynthetic processes (Jabborova et al. 2021; Siswanti and Khairunnisa 2021). Limited direct evidence exists regarding the effects of *P. aphidis* and its secreted compounds on chlorophyll content and photosynthetic activity. Our findings revealed that plants treated with *P. aphidis* extract exhibited significantly elevated chlorophyll content (Fig. 2). The ratio of chlorophyll a to b serves as an indicator of stress responses under conditions such as nitrogen deprivation, light stress, and water stress (Kitajima and Hogan 2003; Zhang et al. 2011). The chlorophyll a to b ratio remained unchanged, suggesting that *P. aphidis* extract treatment did not induce a stress response.

While the treatment did not affect the fresh weight of treated corn plants, treated tomato, and melon plants exhibited a significant increase in fresh weight by 30% and 50%, respectively. Treated tomato and melon plants dry weight increased by twofold compared with controls, the corn dry weight increased by 50% (Fig. 4). The difference in corn fresh and dry weight relative to its control is linked to higher water content in the control plants. The observed lower water content accompanied by higher biomass suggests a trait allowing grain-producing plants to maintain water potential and photosynthesis under drought conditions (Maisura et al. 2014). This suggests that the *P. aphidis* extract may function as a drought protectant. While our primary aim was to enhance the production sustainability, the increase in plant size and biomass may also serve a potentially important role. Dry plant material is a vital resource for fuel and animal feed and with the ever-increasing global population and industry, the demand for these resources is also increasing (Spiertz and Ewert 2009).

An increase in plant biomass, size, and early flowering by PGP stimulants alone does not necessarily imply a positive effect on the crop and the yield, as demonstrated by Zhang and Whiting (2011). Our findings demonstrate that the *P. aphidis* extract positively affected the yield of all the treated plants. The treated tomato plants exhibited unexpectedly higher fruit yield and quality. The fruit matured earlier, with over 60% ripe fruit and yielded nearly 20% more produce in total fruit (Fig. 5). A similar trend was observed in our previous studies utilizing live *P. aphidis* cultures on microtome tomatoes (Shoam et al. 2022). Consequently, we present substantial evidence that the PGP compounds from *P. aphidis* are secreted and align with our previous observation regarding live cultures (Shoam et al. 2022).

While the treated plants yielded large mature fruit, the control melon plants exhibited unripe fruits (Fig. 5). Fruit of treated melon plants exhibited a weight 5 times greater than that of the control plants, with each plant producing twice as many fruits with a diameter more than twice that of the control plants. Corn kernels harvested from treated plants demonstrated a 17% and 20% increase in weight compared with that of the control group (in both fresh and dry states, respectively). Each corn plant produced nearly twice the number of cobs (Fig. 4). Increasing the production of grains has an enormous significance in establishing food security. The potential to achieve this goal through an environmentally sustainable BMO-based approach could significantly reduce the agricultural footprint on the environment, given the extensive land area required for the cultivation of grain crops (Peng et al. 2023).

According to Singh (2018), the monetary value of PGP products, SF are rather costly and bio-fertilizers can reduce the costs and increase the overall gain value (Singh 2018). Our extract is currently in early stages of study and development and it is too soon to assign a value estimate. Nevertheless, based on the aforementioned work, we estimate that it can have a comparable economic expense value to existing bio-fertilizers for the farmer. With that said, our extract enhanced the plant growth and the yield of cereals, *Cucurbits* and *Solanaceae*. Additionally, it enhanced the crop quality of tomatoes, melons, and corn. Thus, *Pseudozyma* extract is rendered a valuable and promising eco-friendly alternative to synthetic PGP products.

An increase in crop yield is often correlated with a decline in produce quality, which encompasses a negative effect on flavor, texture, and aroma (Todeschini et al. 2018). However, it has been demonstrated that grape berries subjected to certain PGP microorganisms during their ripening phase can improve their quality (Ding et al. 2019). Unexpectedly, our research revealed that the fruit of plants treated with our *P. aphidis* extract contained elevated levels of natural sugars, higher concentrations of nitrogen-based compounds, and increased the starch quantity in corn. Thus, indicating an increased fruit sweetness and higher nutritional value (Figs. 6 and 7). Additionally, the fruits harvested from the treated tomato plants demonstrated a greater firmness compared with that of the control plants (Fig. 7). The connection between firmness, fruit quality, and shelf life has been well documented across many cultivars, including tomatoes (Schouten et al. 2007; Matas et al. 2009). Furthermore, fruit firmness is also associated with increased fruit resistance to post-harvest pathogens, such as *B. cinerea,* indicating that firmer fruits are likely to be less susceptible to post-harvest pathogens (Silva et al. 2023). Hence, the treatment with *P. aphidis* extract may potentially extend the shelf life and contribute to food loss reduction.

Although fruit quality, as assessed by sugar concentration, soluble acids, nutrient values, and fruit firmness, exhibited good correlation with sensory flavor attributes. It does not always reflect the subjective flavor quality of the produce as experienced by the consumers (Tandon et al. 2003; Iyer et al. 2012). To complement the fruit quality data, our sensory flavor tests have indicated that the flavor profile of the tomato fruits from the treated plants have consistently received a superior ranking across all the taste parameters (Fig. 7). Flavor parameters of tomatoes from the treated plants, specifically sweetness and acidity, alongside aroma, were ranked slightly higher in comparison to the control group. While the texture, color and overall preference parameters achieved rankings that were 2 ranks higher (Fig. 7). These findings demonstrate the efficacy of *P. aphidis* extract in enhancing produce quality and its potential to improve the flavor.

Our protocol for extract preparation necessitates further optimization to achieve the purification and identification of the PGP compounds and the underlying mechanisms governing their efficacy. This study provides an in-depth analysis of the PGP activity associated with the application of *P. aphidis* extract on crop plants. We demonstrated the capability of compounds secreted by *P. aphidis* to promote plant growth, augment yield, enhance produce quality, and improve flavor. Consequently, this research contributes to the advancement of BMO's eco-friendly agricultural applications and supports the broader objective of global food security.

Plant growth and plant health are also known to have effects on the soil microbiome, particularly the on the rhizosphere (Devi et al. 2020). Studies on the aforementioned subject demonstrate the bio-traits related to plant size and development stage positive effects on the rhizosphere microbiome (Senkovs et al. 2021). Although these aspects were not investigated in our study, the rhizosphere microbiome of the treated plants was probably affected, and this requires a separate dedicated investigation. The phenotype of plants' bio-traits affected by our *P. aphidis* extract was enhanced growth in both size and biomass. These were followed by early development, flowering, fruit formation, maturation, and enhanced crop. In this study, we have established the conditions to cultivate *P. aphidis* and calibrated a methodology for the purpose of PGP-oriented bioactive fraction extraction. We identified the optimal dosage and refined the application method, systematically characterizing its impact on the growth of both juvenile and mature plants, while also demonstrating its influence on crop yield, produce quality, and flavor. Furthermore, we have identified and verified several PGP modes of action utilized by *P. aphidis*. Our extract preparation method should be further fine-tuned to achieve purification and identification of the PGP compounds and the entire underlying mechanisms involved in their activity and efficacy. This study elaborates on the PGP activity of *P. aphidis* extract application on crop plants and demonstrates the ability of compounds secreted by *P. aphidis* to enhance plant growth, increase the yield, produce, flavor quality in fruit, and starch content in corn. Thus, this research contributes to the advancement of BMOs eco-friendly agricultural application and promotes the broader objective of global food security.

## Materials and methods

### PA cultures and plant material

Corn, Raymon melon, and M-82 tomato seeds were sterilized in 3% bleach solution for 5 min, followed by 3 rinses with sterile distilled water (DW), and placed at 4 °C overnight before germination. The treated seeds were coated by dipping in 3 mg/mL extract, while the control seeds were coated with water. The seeds were planted in vermiculite and maintained in a growth chamber at 22 °C under a 10/14 light-dark photoperiod. After a growth duration of 3 to 7 days in the chamber, the seedlings were transferred to a 30 cm pot containing commercial potting soil and placed in a greenhouse under a natural day–night cycle. The greenhouse conditions were 24 °C with 60% RH, and automated irrigation (100% flow for 10 min twice per day). Following the emergence of the first 2 leaves, the treated plants were sprayed bi-weekly with 3 mg/mL extract until fruit initiation, whereas the control plants were sprayed with water.

### PA extract preparation and calibration

Our *P. aphidis* isolate was grown on potato dextrose agar (PDA) plates at 28 °C for 10 days. Two PDA plates were transferred to a 250 mL flask containing 50 mL MS liquid medium. The flasks were incubated in the dark at 28 °C at 175 rpm for 10 days. The cultures were utilized for extract preparation. Extracts were prepared directly from complete culture media along with the cell suspension. The PA flasks were extracted using 4 equivalents (eq) of organic solvents, gradient from hydrophilic (ethanol) to hydrophobic (hexane) on an orbital shaker at 300 rpm for 30 to 180 min. The organic phase was collected, and the solvents were evaporated with a Buchi Rotary evaporator system (R-215). Dry extract was collected, weighed, and resuspended in a 40 eq aqueous solution (70%/30%, DW/extraction solution) (Table S1).

The solvent media combination extracts were screened for optimal PGP performance using a bioassay. Col-0 Arabidopsis seeds were treated with the extract, germinated, and cultivated for 10 days on solid MS. Root and shoot length measurements were conducted to identify the optimal culture medium and solvent combination (Table S1). Next, we examined the antimicrobial efficiency of the leading PGP-performing extract (hexane/acetone) by utilizing an antibiotic paper disc assay. *B. cinerea* and *A. tumefaciens* cultures were incubated for 24 h on PDA and LB plates, then a Whatman paper disc containing extract at a concentration of 1 to 50 mg/mL was placed at the center of the culture plate. The inhibition halo was monitored over a period of 5 consecutive days (Table S2). This methodological approach allowed us to identify an extract demonstrating exclusive PGP activity.

### Pharmacological assays

We cultivated sets of 15 to 30 Col-0 Arabidopsis seeds coated with an extract at different concentrations (0–10 mg/mL) on solid MS media. The root and shoot length of 7-day-old treated seedlings were assessed in order to calculate pharmacological parameters. The lethal dose (LD), effective dose (ED), and toxic dose (TD) are defined as the quantities that produce lethal, beneficial, and adverse effects on a portion of the population, respectively. These parameters were derived following the pharmacological principles established by (Miller and Tainter 2009).

The LD50 parameter represents the dose that results in mortality for 50% of the population. To determine it, the treated germinated seeds count and the mortality of seedlings for a duration of up to 10 days post-germination were normalized following the log method (Singh et al. 2020). The ED50 variable denotes the effective concentrations that exert a positive influence on 50% of the population. The ED50 calculations were done by integrating germination, root, and shoot length parameters of the germinated seedlings. The data were standardized by using the log method and the principles associated with ED and ED50 (Barreto et al. 2002). The TD50 criterion signifies the non-lethal, yet toxic dose that affects 50% of the population. This was computed as the inverse values of ED and ED50 (Jones 1990). The optimal solvent combination of the PGP extract was determined to be 70% hexane and 30% acetone, diluted to a concentration of 3 mg/mL for application as per the pharmacological assessments, and it did not demonstrate any antimicrobial activity *in vitro*.

### Extract application methods and seed coating

The extract was applied to the plants by diffusion spraying and seed coating based on the principles proposed in Chemistry and Technology of Agrochemical Formulations at the application methods chapter (Knowles 2012). Clean uncoated seeds were coated by utilizing an eco-friendly water-based coat using our extract. The sterile seeds were processed with a solution of extract diluted to 3 mg/mL in sterile DW up to 5% (v/v) DW/extract. Coating was achieved by weighing the seeds and dipping them in a solution volume equal to 3 mg/g of total treated seed weight. Submerged seeds were placed in a beaker on an orbital shaker at 50 rpm, under continuous laminar air flow to fully dry. The dry seeds were weighed again to verify the extract coating. The control seeds underwent an identical process that included an equal volume of coating solution composed of water and clean extract solvent (no extract) in the same ratio as was used for the true extract coating.

### Application method screening by a multivariate correlation matrix

Following the establishment of extraction conditions, extract application and dosage, we examined the PGP activity parameters using a multivariate correlation matrix. We selected the parameters exhibiting a correlation coefficient (*r*^2^) greater than 0.8 in relation to the other PGP parameters. The identified parameters were utilized for the extract application assessment of methods, which included seed coating, bi-weekly spraying of the extract on germinated plants, and a combined approach of seed coating and spraying (Figure S1). This systematic screening facilitated the optimization of both the extract composition and its application method for optimal performance.

### Volatile assay

The volatile activity of the *P. aphidis* extract was assessed by germinating sterilized Col-0 Arabidopsis on MS culture media in a split petri dish. One compartment of the split petri dish contained 20 mL MS culture media sown with 10 seeds. The other compartment contained the *P. aphidis* extract, DW, or liquid MS media extract (as used for the fungal cultures). The split dish was incubated for 5 days, and the germination rate was evaluated as a percentage.

### Chlorophyll extraction and measurement

Chlorophyll a and b were extracted from 5-week-old *Solanum lycopersicum* specimens treated with the extract or water following the methodology delineated by Ritchie (2008). The plant material was homogenized in a 90% acetone solution with a Turrax homogenizer. The slurry was centrifuged, and the chlorophyll a and b were quantified using a spectrometer. The concentration was calculated according to the protocol described by Ritchie (2008).

### Auxin extraction and measurement

The auxin concentration was assessed employing a modified Salkowski reagent methodology derived from the approach utilized by Gang et al. (2019). The reagent solution composition was 1 mL of 8% FeCl_3_ in 50 mL of 80% sulfuric acid. For the assay, a 1 mL sample was diluted with 0.5 mL of ethanol and 2 mL of the modified reagent. The reaction mixture was incubated for 30 minutes in dark conditions on an orbital shaker set at 25 rpm. The absorbance was recorded at 530 nm, and indole-3-butyric acid served as the standard for the calibration curve and as a positive control.

### Siderophores extraction and measurement

The presence of siderophores was evaluated using the chrome azurol S (CAS) indicator in liquid MS medium, following the methodology established by Milagres et al. (1999), Qessaoui et al. (2024)), and Verkleij (1992). The *P. aphidis* extract was incubated in the presence of the CAS indicator reagent and subsequently analyzed with an Alginet Biotec 800 TS spectrometer, carboxylate was utilized as the positive control.

### Plant physiological parameters measurement

Germination was evaluated as the percentage of germinating seedlings per total sown seeds and evaluated after 10 to 14 days, after no new germinating seedlings sprouted. Flowering was evaluated as the percentage of flowering plants out of all the plants from the time stamp a plant started to develop flowers until reaching full mature bloom. Shoot height was measured on 5-week-old plants and mature plants at harvest time. Leaves length and the number of leaves of 5-week-old plants were manually evaluated. Mature plant size was evaluated by Fiji ImageJ. The dry weight of mature plants and 100 corn kernels was evaluated by fully drying them in a 45 °C oven.

### Tomato ripening and firmness determination

Tomato fruit ripening was visually assessed following the established color ripening scale as portrayed by Takizawa et al. (2014). In the assessment of fruit ripeness, 5 distinct stages of maturation were identified; green-unripe, which refers to the unripe green fruit, breaker, which denotes tomatoes that have begun to exhibit a slight red hue indicative of ripening, turning, characterized by a significant portion of the fruit transitioning to a pink/red coloration, light red, where the entire fruit appears pale red, and red, which signifies the fruit is fully ripe and exhibits a deep red color. The evaluation of ripening stages was conducted visually, and all harvested tomato fruits were meticulously examined on a per-plant and per-treatment basis. The firmness of the harvested tomatoes was quantitatively assessed with a Shore analog durometer, as noted by Fei et al. (2018). The classification of fruit firmness was categorized based on a spectrum of durometer units (DU); rank 1 indicates very soft (0–10 DU), rank 2 designates soft (11–20 DU), rank 3 refers to flexible (21–30 DU), rank 4 represents firm (31–50 DU), and rank 5 signifies hard fruit (51–100 DU).

### TSS, TA, nitrogen content, and sensory test

TSS content of tomato and melon juice was determined utilizing the methodology established by Yadav et al. (2021a, 2021b). We extracted 3 mL of juice from 5 individual ripe tomato fruits and 3 melons; the extracted juice was subjected to filtration through 5 layers of cheesecloth and subsequently diluted to a 1:1 ratio with DW. A volume of 1 mL was then analyzed using a fruit juice refractometer, with results reported in degrees Brix (°Brix). The TA and titratable nitrogen content of the fruit juice were assessed employing the protocols published by the research teams of Heeb et al. (2005) and Majidi et al. (2011). To determine TA, 5 mL of tomato juice was titrated with 0.1N sodium hydroxide (NaOH) until a pH 8 was achieved.

For the quantification of total nitrogen content, the titrated samples were treated with formaldehyde and further titrated with 1 N NaOH to reach pH 8. The results of TA are conveyed in terms of citric acid equivalents. The sensory evaluation of mature ripe tomato fruit was conducted following the methodology utilized by Heeb et al. (2005). Fruits harvested from treated and control mature plants were labeled with random letters to ensure anonymity. In the sensory evaluation, a group of 32 individuals was presented with a survey designed to rank sensory attributes such as sweetness, sourness, texture, color, and overall preference. The evaluation parameters were assigned ranks ranging from 1, indicating the lowest preference, to 10, signifying the highest preference.

### Corn starch measurement

Corn starch quantity of kernels from treated and control plants was extracted and measured according to the colorimetric method used by the research teams of McGrane (2002) and Saibine (2008). The kernels were fully dried, 1 g of kernels was weighed and powdered, 100 mg of the powder was dissolved in 4 mL DMSO on a heating block at 85 °C for 15 min. Potassium iodide (PI) reagent was prepared by mixing 0.0025 mol/L iodine with 0.0065 mol/L. The dissolved starch slurry was diluted to 50 mL with DW on a stirrer. For the reaction, 5 mL of PI reagent was added to the starch extract solution, and data were obtained by measuring the absorption at 600 nm with Alginet Biotec 800 TS spectrometer (McGrance et al. 1998). Calibration curve was done with analytical grade corn starch obtained from Sigma-Aldrich in concentrations of 0, 10, 25, 50, 75, and 100 mg, resulting *r*^2^ = 0.98.

### Statistical analysis

Statistical analyses were conducted with SAS JMP 11 software for multiple comparisons, multivariate correlation, matrix analyses, and Student's *t*-test. Data were analyzed according to the experiments at *α* = 0.05.

## Supplementary Material

kiag079_Supplementary_Data

## Data Availability

The data are available in the article and in its online supplementary material.
