# Supplementary material for: Make it grow: Pseudozyma aphidis extract promotes plant growth
Source: Plant Physiol. 2026 Apr 29;200(4):kiag079. doi: 10.1093/plphys/kiag079 (PMC13127791; doi:10.1093/plphys/kiag079)
Supplement: kiag079_Supplementary_Data [file kiag079_supplementary_data.pdf]

Supplementary

Table S1. Root length and shoot length.

Treatment	Root length (cm)	Shoot length (cm)
**Control DW**	**1.21 ± 0.03 h**	**0.34 ± 0.1 h**
**Control PDB**	**1.21 ± 0.02 h**	**0.35 ± 0.01 h**
**Control MS**	**1.18 ± 0.03 h**	**0.41 ± 0.02 fg**
**PDB Ethanol**	**1.5 ± 0.07 cd**	**0.55 ± 0.03 abc**
**MS Ethanol**	**1.31 ± 0.05 g**	**0.38 ± 0.02 gh**
**PDB Chloroform**	**1.5 ± 0.04 cd**	**0.45 ± 0.02 ef**
**MS Chloroform**	**1.44 ± 0.04 f**	**0.53 ± 0.02 cd**
**PDB Ethanol 70% Acetone 30%**	**1.48 ± 0.02 cde**	**0.6 ± 0.01 a**
**MS Ethanol 70% Acetone 30%**	**1.45 ± 0.03 ef**	**0.5 ± 0.1 de**
**PDB Acetone**	**1.45 ± 0.03 f**	**0.54 ± 0.02 bcd**
**MS Acetone**	**1.48 ± 0.02 def**	**0.52 ± 0.02 cd**
**PDB Hexane 70% Acetone 30%**	**1.52 ± 0.02 c**	**0.58 ± 0.02 ab**
**MS Hexane 70% Acetone 30 %**	**1.56 ± 0.02 a**	**0.6 ± 0.01 a**
**PDB Hexane**	**1.3 ± 0.01 g**	**0.5 ± 0.01 de**
**MS Hexane**	**1.61 ± 0.01 a**	**0.56 ± 0.02 abc**

Col-0 arabidopsis plants treated with 3-4 mg/ml *P. aphidis* extracts of deferent solvent and culture media combination (MS and PDB). The solvents were selected by their hydrophilic/phobic characteristics from ethanol to hexane. Root and shoot length measurements were obtained from 7-day old treated seedlings. Mean and standard error accompanied by connecting letters represent significant deference calculated with students t test, α=0.05; n=50 plants per media/solvent combination treatment.

Table S2. ***P. aphidis*** **extract test for antimicrobial activity.**

Extract	Concentration mg/ml	Bo.5 halo	*A. tumefaciens* halo
**MS Hexane 70% Acetone 30%**	**1**	**0 ± 0**	**0 ± 0**
	**5**	**0 ± 0**	**0 ± 0**
	**10**	**0 ± 0**	**0 ± 0**
	**25**	**0 ± 0**	**0 ± 0**
	**50**	**0 ± 0**	**0 ± 0**

MS hexane acetone solvent combination tested for antimicrobial activity against *B. cinerea* (Bo.5) and *A. tumefaciens* invitro. Agrobacterium and Botrytis were grown on solid medium for 24 hours and then a disc with extract was placed in the centre of the petri dish. The disk was loaded with 1mg/ml to 50 mg/ml extract and the inhibition halo was measured after 5 days.

Table S3. **Germination time of corn, melon and tomato plants following *P. aphidis* extract treatment.**

Plant	Group	Gemination day (over 50% germination)	Gemination day (Complete germination 75-100%)
Maize	Control	10 ± 1.3 b	14 ± 1.145 b
	Treatment	8 ± 1.12 a	12 ± 1.07 a
Melon	Control	11 ± 0.95 b	13 ± 0.78 b
	Treatment	9 ± 0.84 a	11 ± 0.52 a
Tomato	Control	11 ± 1.3 b	14 ± 1.56 b
	Treatment	8 1.12 a	11 ± 1.54 a

Seeds coated with *P. aphidis* extracts germination time from sawing to 50% germination and complete germination (75% to 100%). Mean and standard error accompanied by connecting letters represent significant deference calculated with students t test, $\alpha=0.05$; n=80 seeds for each plant group and treatment.

a

Parameter	Number of segments												
Number of leaves	1.000	Number of leaves											
Green flowers	0.081	0.074	Green flowers										
Mature flowers	0.000	0.000	0.995	Mature flowers									
Fruit stage 1	0.305	0.311	0.000	0.000	Fruit stage 1								
Fruit stage 5	0.854	0.857	0.000	0.000	0.756	Fruit stage 5							
Leaves width	0.092	0.086	1.000	0.994	0.000	0.000	Leaves width						
Leaves length	0.070	0.064	1.000	0.996	0.000	0.000	1.000	Leaves length					
Height	0.714	0.719	0.000	0.000	0.885	0.974	0.000	0.000	Height				
Mature fruit weight	0.000	0.000	0.000	0.000	0.730	0.104	0.000	0.000	0.326	Mature fruit wight			
Total fruit weight	0.000	0.000	0.000	0.000	0.891	0.376	0.000	0.000	0.577	0.961	Total fruit weight		
Shoot dry weight	0.937	0.939	0.000	0.000	0.619	0.982	0.000	0.000	0.914	0.000	0.195	Shoot dry weight	
Shoot fresh weight	0.672	0.667	0.792	0.727	0.000	0.189	0.800	0.786	0.000	0.000	0.000	0.371	Shoot fresh weight

Correlation (r^2)
1.000
0.850
0.750
0.650
0.550
0.350
0.195
0.000

b

Number of leaves	Number of segments	1.000	Total fruit weight	Mature fruit weight	0.961
Leaves width	Green flowers	1.000	Shoot dry weight	Number of leaves	0.939
Leaves length	Green flowers	1.000	Shoot dry weight	Number of segments	0.937
Leaves length	Leaves width	1.000	Shoot dry weight	Height	0.914
Leaves length	Mature flowers	0.996	Total fruit weight	Fruit stage 1	0.891
Mature flowers	Green flowers	0.995	Height	Fruit stage 1	0.885
Leaves width	Mature flowers	0.994	Fruit stage 5	Number of leaves	0.857
Shoot dry weight	Fruit stage 5	0.982	Fruit stage 5	Number of segments	0.854
Height	Fruit stage 5	0.974	Shoot fresh weight	Leaves width	0.800

c

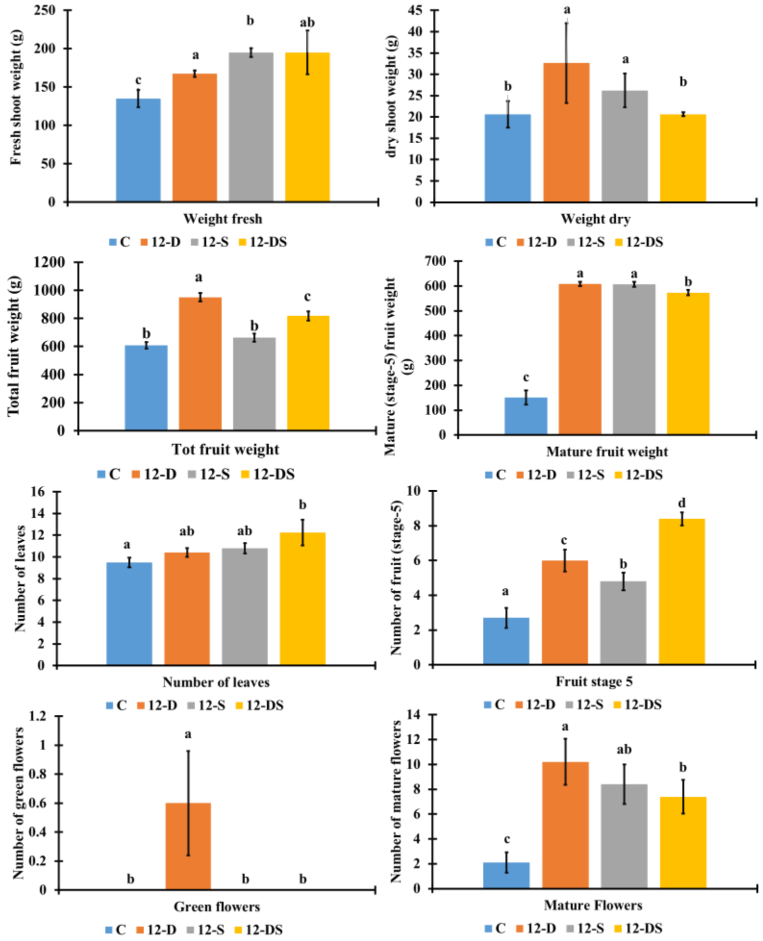

Fig. S1 Multivariate correlation matrix of all the *P. aphidis* (PA12) treatments. a. Multivariate matrix, green indicates high correlation between plant parameters that across all treatments and red indicates low to no correlation between the parameters across all treatments. b. summary table of parameters with high correlation across all treatments that indicate plant growth promoting activity. Parameters with r^2 values higher than 0.8 were selected. c. The blue bars labelled C, represent control, orange bars labelled 12-D represent seed coating treatment, grey bars labelled 12-S show spray treatment and yellow bars labelled 12-DS show combined seed coating and spray treatments data. Comparison of treatment methods on all significant parameters (r^2>0.8), the column bars represent the average values, error bars represent Standard Error. Connecting letters adjacent to the data bars are Significance ranking as obtained by Students T-test, α=0.05.
